# The influence of subretinal injection pressure on the microstructure of the monkey retina

**DOI:** 10.1371/journal.pone.0209996

**Published:** 2018-12-31

**Authors:** Kosuke Takahashi, Yuki Morizane, Toshio Hisatomi, Takashi Tachibana, Shuhei Kimura, Mio Morizane Hosokawa, Yusuke Shiode, Masayuki Hirano, Shinichiro Doi, Shinji Toshima, Ryoichi Araki, Hiroshi Matsumae, Yuki Kanzaki, Mika Hosogi, Atsushi Yoshida, Koh-Hei Sonoda, Fumio Shiraga

**Affiliations:** 1 Department of Ophthalmology, Okayama University Graduate School of Medicine, Dentistry, and Pharmaceutical Sciences, Okayama, Japan; 2 Department of Ophthalmology, Graduate School of Medical Sciences, Kyushu University, Fukuoka, Japan; 3 Research and Development Division, Santen Pharmaceutical Co., Ltd., Nara, Japan; Massachusetts Eye & Ear Infirmary, Harvard Medical School, UNITED STATES

## Abstract

**Purpose:**

To investigate the influence of subretinal injection pressure on the microstructure of the retina in a monkey model.

**Methods:**

After vitrectomy, balanced salt solution was injected subretinally into one eye each of four cynomolgus monkeys while controlling the injection pressure. Initially, a pressure of 2 psi was used, and this was gradually increased to determine the minimum required pressure. Subsequent injections were performed at two pressures: minimum (n = 13) and high (n = 6). To compare the influence of these injection pressures on retinal structure, optical coherence tomography (OCT) was performed before surgery and every week afterwards. The monkeys were euthanized and their eyes were enucleated at 1 or 6 weeks after the injections. The eyes were processed for light microscopy and transmission electron microscopy (TEM) as well as for TdT-mediated dUTP nick end labeling.

**Results:**

The minimum pressure required to perform subretinal injection was 6 psi. After injection at this pressure, both OCT and microscopy showed that the retinal structure was well-preserved throughout the experimental period at all injection sites. Conversely, after injection at high pressure (20 psi) OCT images at all injection sites showed disruption of the ellipsoid zone (EZ) after 1 week. Microscopy indicated damage to the photoreceptor outer segment (OS) and stratification of the retinal pigment epithelium (RPE). After 6 weeks, OCT demonstrated that the EZ had become continuous and TEM confirmed that the OS and RPE had recovered. Photoreceptor apoptosis was absent after subretinal injection at both pressures.

**Conclusions:**

The retinal damage caused by subretinal injection increases depending on pressure, indicating that clinicians should perform subretinal injection at pressures as low as possible to ensure safety.

## Introduction

Surgical subretinal injections are used to displace subretinal hemorrhages [1–4], deliver gene therapy for retinal degeneration [5–8], perform macular translocation in patients with age-related macular degeneration [9,10], remove hard foveal exudates [11], and resolve diffuse diabetic macular edema during planned foveal detachment procedures [12,13]. To ensure that visual function is preserved after such procedures, clinicians must not damage the macula, and a safe and reliable technique for subretinal injection must therefore be developed.

To deliver a subretinal injection to a patient with an attached retina, the injection pressure applied must exceed the adhesion force between the retina and retinal pigment epithelium (RPE) [14–16]. However, when a liquid is injected into the subretinal space, the stream may physically damage the structure of the outer retina and the RPE [17]. According to Poiseuille’s law, when a liquid of consistent viscosity is injected through a cannula with a consistent diameter, the flow rate is determined by the injection pressure [14]. However, the relationship between injection pressure and consequent retinal damage has not been sufficiently elucidated.

On the basis of Laplace's law, patients with an attached retina require the highest injection pressure to initiate retinal detachment, and this required pressure decreases as the area of retinal detachment expands [18]. Therefore, in the present study, we investigated the effect of injection pressure, which is required to initiate retinal detachment, on the microstructure of the monkey retina using *in vivo* optical coherence tomography (OCT), light and electron microscopy, and TdT-dUTP terminal nick-end labeling (TUNEL).

## Methods

### Ethics statement

All animal experiments were reviewed and approved by the Institutional Animal Care and Use committee (IACUC) of Santen Pharmaceutical Co., Ltd. (approval no. DR-2016-0157). Male cynomolgus monkeys (*Macaca fascicularis*) aged 3–4 years and weighing 3.0–5.0 kg were purchased from Eve Bioscience Ltd. (Wakayama, Japan). The animal welfare and steps taken to ameliorate suffering were in accordance with Guidelines for Proper Conduct of Animal Experiments (Science Council of Japan) and the recommendations of the Weatherall report on the use of non-human primates in research. Because there was no group breeding environment in the breeding facility, the animals were housed individually in stainless steel cages (width: 47 cm, depth: 89 cm, height: 76 cm) at the animal facility of Santen Pharmaceutical Co., Ltd., where the environmental conditions were as follows: room temperature, 24°C; relative humidity, 60%; illumination, 12-hour lighting (7 a.m. to 7 p.m.) at 300 lux. The cage sizes followed the criteria of Institutional for Laboratory Animal Research and the IACUC approved the individual housing of monkeys and the cage sizes. The animals were fed 100 g/animal/day of pellet food for monkeys (Monkey Bit; Nosan Corporation, Yokohama, Japan). Tap water from a feed-water nozzle was supplied ad libitum. During subretinal injection and OCT examination, monkeys were anesthetized by intramuscular injection of ketamine hydrochloride (10 mg/kg). Respiratory rate was monitored frequently and used to maintain adequate anesthesia using ketamine. Topical drops of oxybuprocaine were used for analgesia. Topical drops of 0.5% phenylephrine hydrochloride and 0.5% tropicamide were used for mydriasis. The monkeys were sedated and humanely euthanized by intravenous pentobarbital by a licensed veterinarian, either 1 week or 6 weeks after subretinal injections. Confirmation of death was determined by monitoring for absence of pulse, respiration, and neural reflexes. The eyes were enucleated immediately after euthanasia, either 1 week or 6 weeks after subretinal injections.

### Subretinal injection with local removal of the internal limiting membrane

**Fig 1:**
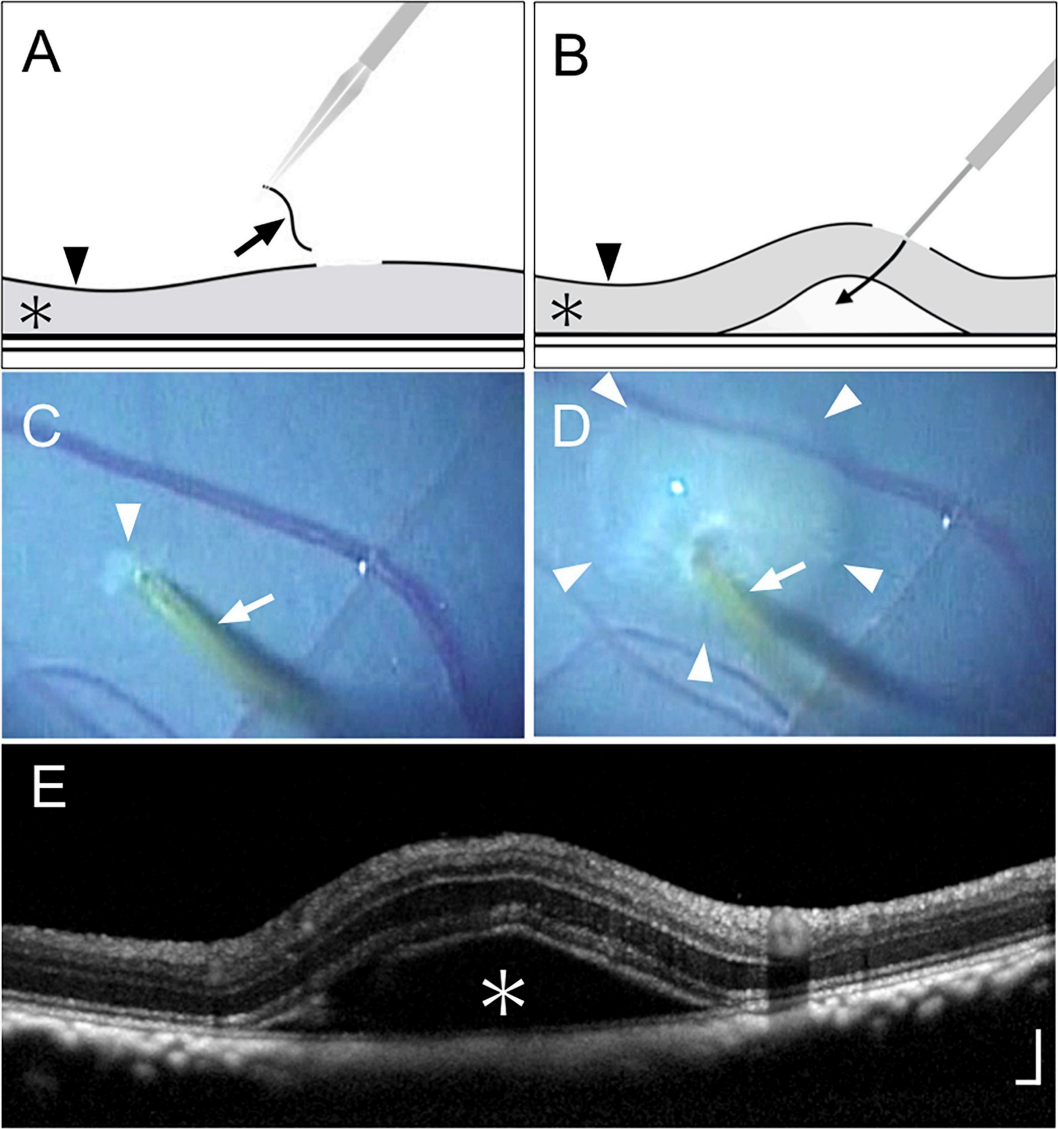
Local removal of internal limiting membrane and subretinal injection of balanced salt solution. (A) Schematic drawing of local internal limiting membrane (ILM) removal showing the removed ILM (arrow), retina (asterisk), and intact ILM (arrowhead). (B) Schematic drawing of the subretinal injection procedure. Balanced salt solution (BSS) was injected with a 38-gauge cannula by placing the cannula tip in contact with the retinal nerve fiber layer. The arrow indicates the flow of the injected BSS; the asterisk and arrowhead indicate the retina and ILM, respectively. (C) Surgical photograph after local ILM removal. The arrow indicates the 38-gauge cannula; the arrowhead indicates the area of peeled ILM. (D) Surgical photograph during subretinal injection. The arrow indicates the 38-gauge cannula; arrowheads indicate the area of retinal detachment due to subretinal injection of BSS. (E) Optical coherence tomography 30 minutes after subretinal injection. The asterisk indicates retinal detachment caused by the procedure. Scale bar = 200 μm.

In total, four monkeys were subjected to surgical subretinal injections in one eye. Specifically, transconjunctival, 25-gauge, 3-port, pars plana vitrectomies were performed using a commercially available vitrectomy machine (Accurus; Alcon Laboratories Inc., Fort Worth, TX, USA). Each injection site was at a mid-peripheral location within the eye. The internal limiting membrane (ILM) was then removed locally over approximately 1/4 of the disc area at each injection site, and balanced salt solution (BSS) was injected using a 38-gauge cannula (MedOne, Sarasota, FL, USA) until the area of retinal detachment had expanded to approximately one disc area (Fig 1), as described in our previous report [19]. By using this method, the BSS could be injected subretinally by placing the cannula tip in contact with the retinal nerve fiber layer exposed by ILM removal; thus, penetration of the retina with the cannula was unnecessary.

### Investigation of minimum required injection pressure and establishment of experimental groups

To ensure a constant pressure, subretinal injection was performed using the Viscous Fluid Control System (VFC; Alcon Laboratories Inc.), which allows the operator to raise the injection pressure from 2 psi to 80 psi in increments of 2 psi. Therefore, to identify the minimum required injection pressure, we began at 2 psi and increased the pressure until subretinal injection became possible. After the minimum required injection pressure was established (6 psi), we performed subretinal injection at this pressure at three or four sites in each eye (minimum-pressure group), for a total of 13 sites (S1–S4 Figs). To compare the influence of the procedure on the retina at different pressures, we also performed high-pressure subretinal injection (20 psi) at one or two points in each eye (high-pressure group), for a total of six sites (S1–S4 Figs). We also performed local removal of the ILM, but not subretinal injection, at a total of three sites in two eyes (control group). We identified all injection sites after the surgery by using a recorded surgical video and conducted subsequent investigations.

### Optical coherence tomography

**Fig 2:**
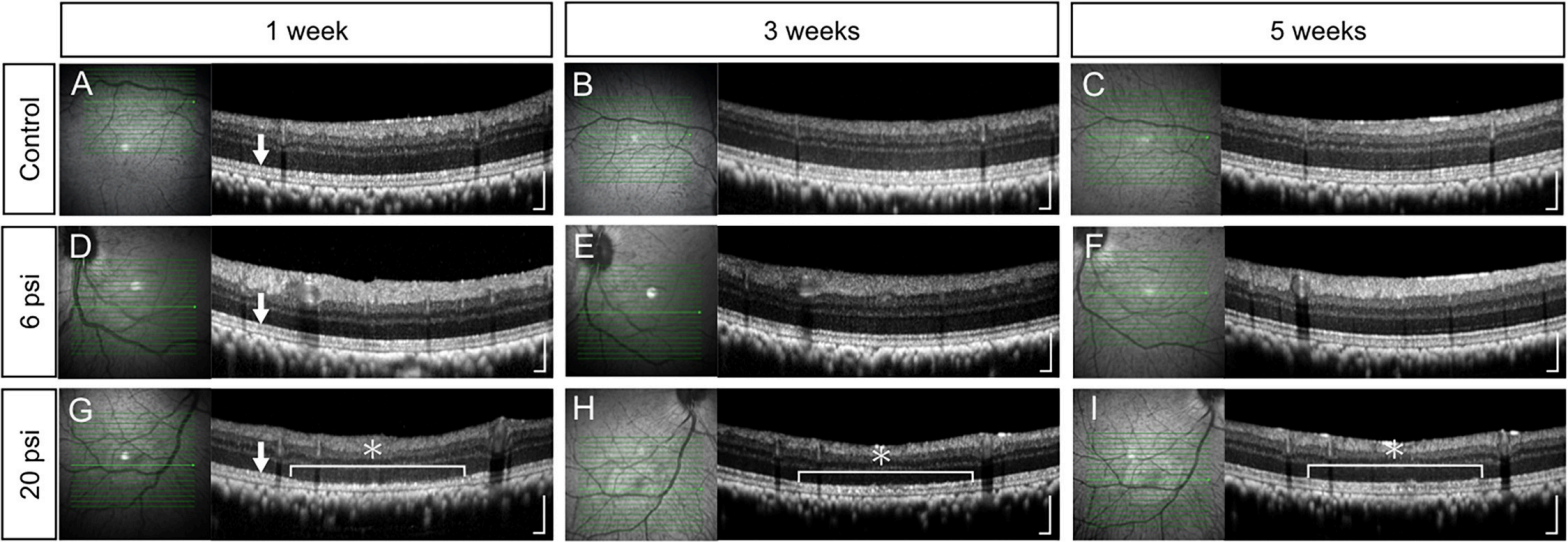
Optical coherence tomography images of monkey retina after subretinal injection of balanced salt solution. Optical coherence tomography (OCT) images of both control (no injection of balanced salt solution; BSS) and minimum-pressure (BSS injection at 6 psi) groups show a well-preserved retinal structure throughout the experimental period, including continuity of the ellipsoid zone (EZ) (A to F). OCT image from the high-pressure group (BSS injection at 20 psi) show EZ disruption (asterisk in G) at 1 week after the injection (G). At 3 weeks after the injection (H), OCT image from the high-pressure group shows partial recovery of the EZ (asterisk). The EZ finally became continuous (asterisk in I) at 5 weeks after the injection. Arrows in A, D, and G indicate the EZ. Scale bars = 200 μm.

OCT (Spectralis; Heidelberg Engineering GmbH, Heidelberg, Germany) was performed before surgery and every week after surgery until the 5^th^ week. OCT images were first obtained 1 week after injection because this was the time point when postoperative inflammation was reduced and clear OCT images were stably obtained in all cases. To improve reproducibility, the exact injection site and area of retinal detachment were identified based on the surgical video (Fig 1D) and infrared fundus images (Fig 2 and S1–S4 Figs).

### Light and transmission electron microscopy

Two eyes, which were enucleated at either 1 week (S3 Fig) or 6 weeks (S1 Fig) after injections, were investigated with light and transmission electron microscopy (TEM). The eyes were fixed in a solution of 1% glutaraldehyde and 1% paraformaldehyde in phosphate-buffered saline. The specimens were post-fixed in veronal acetate buffer containing osmium tetroxide (1%), dehydrated in ethanol and water, and then embedded in Epon resin (Epon 812 Resin; TAAB Laboratories, Aldermaston, UK). The eyes were cut into 1-μm-thick sections, stained with toluidine blue, and observed by light microscopy. Tissue sections were prepared based on a surgical video and retinal blood vessels of enucleated eyeballs. For TEM, ultrathin sections were cut from the Epon resin blocks and mounted on copper grids. The specimens were observed by using an H-7770 transmission electron microscope (Hitachi, Tokyo, Japan).

### TdT-dUTP terminal nick-end labeling

Apoptosis of photoreceptor cells was investigated using TUNEL staining. For this analysis, two eyes were enucleated at 1 week (S4 Fig) and 6 weeks (S2 Fig) after injections and were fixed by using a mixture of methanol and formalin (Superfix; Kurabo, Osaka, Japan) at room temperature for the first 2.5 hours and at 4°C for the next 3 days. The eyes were then embedded in paraffin and cut into 3-μm sections. Tissue sections were prepared based on a surgical video and retinal blood vessels of enucleated eyeballs. TUNEL staining was performed by using an *in situ* apoptosis detection kit (Takara Bio Inc., Shiga, Japan), in accordance with the manufacturer’s protocol.

## Results

### Minimum pressure required for subretinal injection with local removal of ILM

The minimum pressure required for subretinal injection was 6 psi, and the procedure was thus performed at this pressure in the minimum-pressure group. In the high-pressure group, subretinal injection was performed at 20 psi, based on the upper limit of the range of clinically-used injection pressures for gene therapy [15].

### Influence of minimum and high-pressure injection on the structure of the retina

*In vivo* OCT examination revealed that there were no defects in the ellipsoid zone (EZ) at any of the 13 injection sites in the minimum-pressure group. Additionally, the retinal structure was identical between the minimum-pressure group and the control group, indicating that subretinal injection at 6 psi did not damage the retina (Fig 2A–2F and S1–S4 Figs). Conversely, in the high-pressure group, all six injection sites showed defects in the EZ at 1 week after injection (Fig 2G and S1–S4 Figs). These defects had begun to resolve at 3 weeks after injection (Fig 2H, S1 and S2 Figs) and had almost completely recovered at 5 weeks after injection (Fig 2I, S1 and S2 Figs).

**Fig 3:**
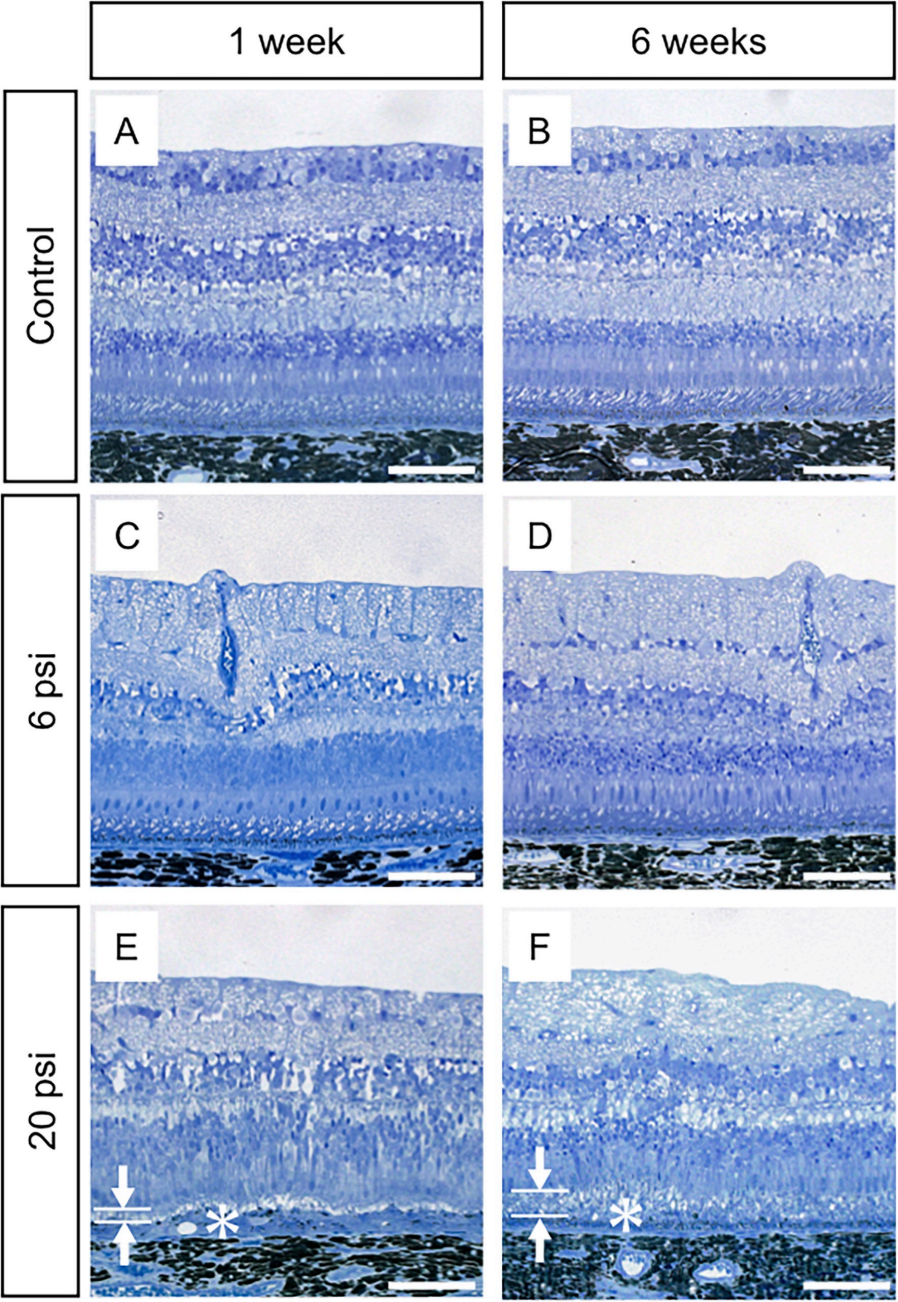
Light microscopy images of monkey retina after subretinal injection of balanced salt solution. The retinal structures of both the control (no injection of balanced salt solution; BSS) and the minimum-pressure (BSS injection at 6 psi) groups are well-preserved at 1 week (A and C) and 6 weeks (B and D) after injection. The high injection pressure group (BSS injection at 20 psi) shows thinning of the photoreceptor outer segment layer (OS, arrows in E) and thickening of the retinal pigment epithelium (RPE) layer (asterisk in E) at 1 week after injection (E), while the photoreceptor cells are well-preserved. At 6 weeks after injection, the high-pressure group shows restoration of the OS (arrows in F) and flattening of the RPE (asterisk in F). Scale bars = 100 μm.

**Fig 4:**
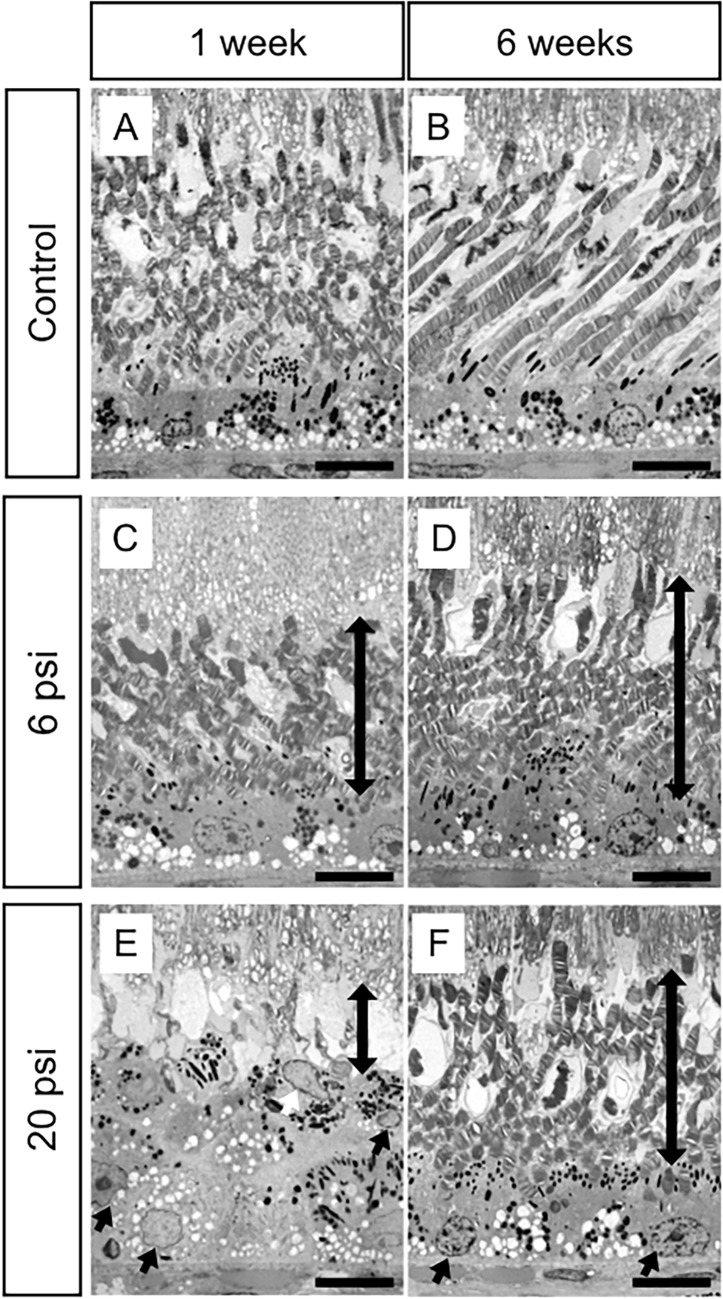
Transmission electron microscopy images of monkey retina after subretinal injection of balanced salt solution. Retinal structures of the control group (no injection of balanced salt solution; BSS) are well-preserved at 1 week (A) and 6 weeks (B) after injection. The minimum-pressure group (BSS injection at 6 psi) shows a shorter photoreceptor outer segment (OS) than the control group at 1 week after injection (C, bidirectional arrow). The OS is restored at 6 weeks after injection (D, bidirectional arrow). The retinal pigment epithelium (RPE) is well-preserved throughout the experimental period (C and D). Conversely, the high-pressure group (BSS injection at 20 psi) shows degeneration of the OS (bidirectional arrow in E) and migration of RPE cells (arrows in E), leading to multiple RPE layers at 1 week after injection. Regeneration of the OS (bidirectional arrow in F) and flattening of the RPE (arrows in F) were observed at 6 weeks after the injection. Scale bars = 10 μm.

To further investigate our OCT findings that damaged EZ was observed 1 week after injection and had almost completely recovered by 5 weeks after injections (Fig 2G, Fig 2I, S1 and S2 Figs), eyeballs were enucleated at 1 or 6 weeks after surgery. We then examined the influence of subretinal injection on retinal structure by using light microscopy and TEM. In the minimum-pressure group, light microscopy showed normal retinal structure at both 1 and 6 weeks after injections (Fig 3C and 3D); this result was similar to that in the control group (Fig 3A and 3B). However, TEM analysis showed that the photoreceptor outer segment (OS) lengths were slightly shorter in the minimum-pressure group than in the control group at 1 week after injections (Fig 4A and 4C). At 6 weeks after the injections, the OS lengths had recovered and were identical to that of the control group (Fig 4B and 4D). No RPE damage was observed by either light microscopy or TEM. In the high-pressure group, disappearance of the OS and stratification of the RPE were observed at 1 week after injections by both light microscopy and TEM (Figs 3E and 4E). These defects had partially resolved at 6 weeks after injections, when reconstruction of the OS and flattening of the RPE were observed (Figs 3F and 4F).

### Photoreceptor cell death after subretinal injection at 6 psi and 20 psi

**Fig 5:**
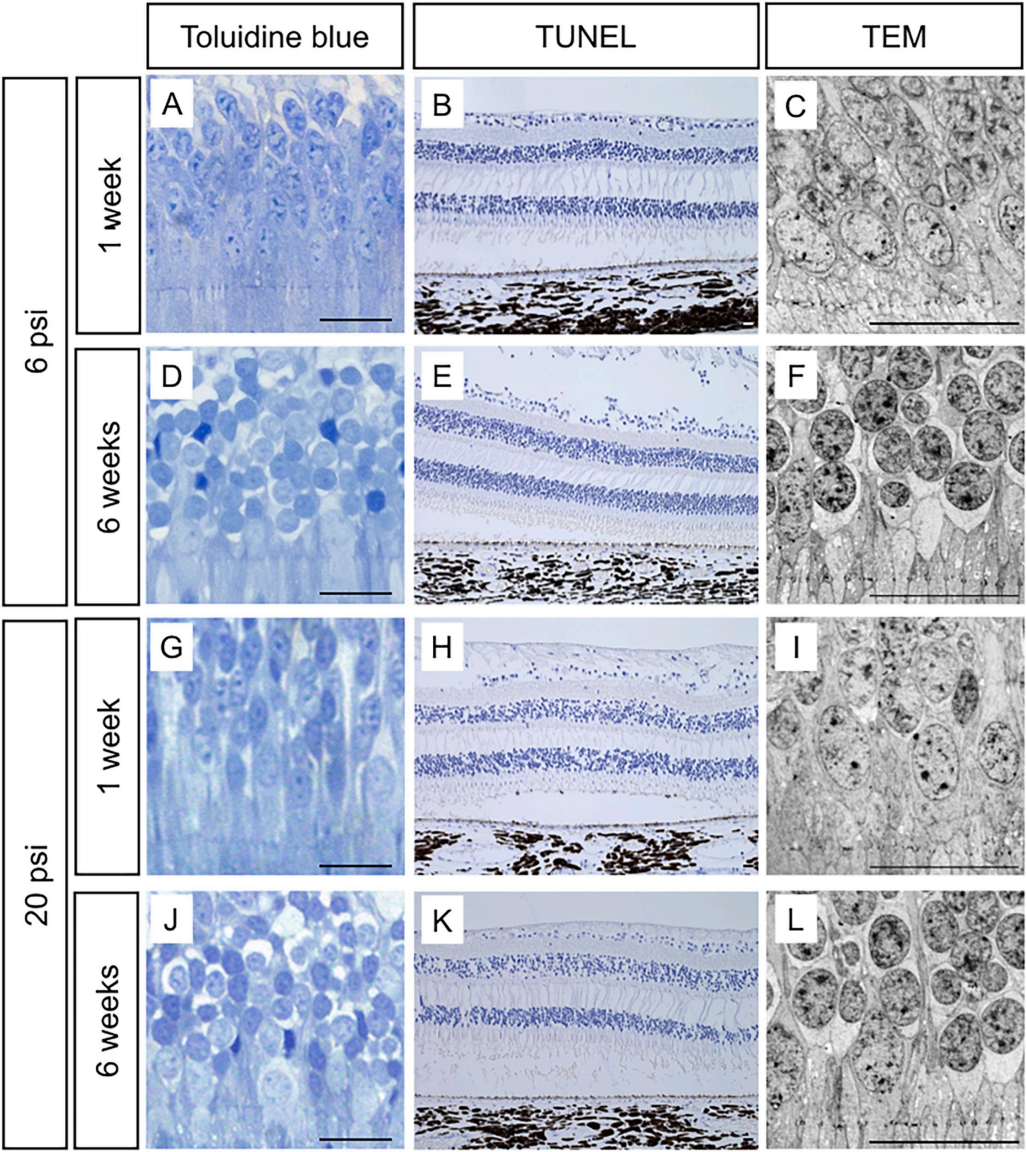
The effect of subretinal injection on photoreceptor cells. In the minimum-pressure (injection at 6 psi with balanced salt solution; BSS) and high-pressure (BSS injection at 20 psi) groups, both light microscopy images with toluidine blue staining and transmission electron microscopy (TEM) images show normal morphology of photoreceptor cells at 1 week (A, C, G and I) and 6 weeks (D, F, J, and L) after injection. TdT-dUTP terminal nick-end labeling (TUNEL) showed no positive photoreceptor cells in either the minimum-pressure or high-pressure groups throughout the experimental period (B, E, H, and K). Black and white scale bars = 20 μm. Positive control of TUNEL staining is shown in S5 Fig.

Light microscopy images with toluidine blue staining showed normal morphology of photoreceptor cells at 1 and 6 weeks after injections in both groups (Fig 5A, 5D, 5G and 5J). TUNEL staining showed no positive cells at either 1 or 6 weeks after injections in either group (Fig 5B, 5E, 5H and 5K). Consistent with this result, TEM images showed that no chromatin condensation or nuclear fragmentation, which are characteristic features of apoptotic cell death, had occurred in the photoreceptor cells of either group at either time point (Fig 5C, 5F, 5I and 5L).

## Discussion

In the present study, we showed that the photoreceptor cells and the RPE of the monkey retina can be damaged by subretinal injection. To our knowledge, this is the first report to reveal that higher injection pressure causes greater retinal damage. Our OCT (Fig 2D) and TEM (Fig 4C) results show that the retinal structure was relatively well-preserved when subretinal injection was applied at 6 psi. Conversely, severe EZ disruption was observed by OCT following subretinal injection at 20 psi (Fig 2G). However, the EZ had become continuous at 6 weeks after 20 psi subretinal injection (Fig 2I). Reflecting the OCT results, TEM images showed severe OS damage (Fig 4E) after 20 psi subretinal injection. However, as shown in Fig 5, the photoreceptor cells were intact; thus, the OS had recovered (Fig 4D and 4F) at 6 weeks after subretinal injection. These results are in agreement with the OS recovery observed after experimental short-term retinal detachment in monkey eye [20,21] and are similar to the OS recovery observed after retinal reattachment in patients with retinal detachment [22–24]. Considering that the severity of retinal damage was dependent on the injection pressure, subretinal injection should be performed at pressures as low as possible to ensure safety.

The resistance associated with subretinal injection arises from the stiffness of the neural retina tissue and the adhesion force between the retina and the RPE; thus, the injection pressure must exceed this resistance [14,15]. Alternatively, the resistance caused by tissue stiffness can be eliminated by puncturing the neural retina with the injection cannula. However, this can damage the retina, RPE, and choroidal vessels, thereby causing serious complications, such as subretinal bleeding and choroidal neovascularization. Most tissue resistance within the neural retina originates from the ILM [25]. Therefore, to resolve the problems of subretinal injection, we recently attempted local removal of the ILM at the subretinal injection site [19]. This method enabled us to perform subretinal injection at a much lower pressure (6 psi) without puncturing the retina. Based on this finding, the present study investigated subretinal injection at the site of local ILM removal, revealing that subretinal injection could be performed at a pressure of 6 psi without puncturing the retina. These results are consistent with our recent findings in the human eye [19] indicating that local removal of the ILM eliminates the resistance caused by the tissue stiffness of the neural retina, thus removing the need for retinal puncture.

Although we focused on injection pressure in the present study, several other factors should be considered with respect to the retinal damage caused by subretinal injection. The first is the volume of the liquid to be injected under the retina, which differs depending on the aim and content of the subretinal injection (tPA [2,4,26], adenovirus vector [5,6,8], cell suspension [27–29], or BSS [9–13]). It has been reported that excessive stretching of the retina by the injected liquid can cause photoreceptor cell death by extension stress [15]. Secondly, the duration of retinal detachment should be considered. Several studies have reported that, in cases of long-term retinal detachment, apoptotic cell death of photoreceptor cells occurs due to a deficiency of nutrients from the choroid [20,30–32]. Thirdly, if the subretinal injection location includes the fovea, a macular hole may occur due to the injection pressure [6,7,33]. Finally, the presence or absence of adhesion between the retina and the RPE can influence the risk of retinal damage after subretinal injection. In cases of gene therapy or cell transplantation therapy to treat retinal degenerative diseases, subretinal injection must sometimes be performed at a site of retinal–RPE adhesion [5–8,15,34]. In such cases, the possibility of damaging the retina and RPE increases because higher injection pressures must be applied to exceed the adhesive force.

This study has several important limitations. Firstly, it is unclear whether our results from monkey eyes can be generalized to humans. Secondly, although we revealed the effects of subretinal injection outside the macula, the effects of subfoveal injection on the fovea remain unknown. In the present study, we performed subretinal injections at several mid-peripheral locations at different injection pressures in the same eye because we wished to examine the influence of injection pressure on the retina by establishing identical injection conditions. However, both the presence or absence of the foveal depression and the proportion of cone and rod photoreceptor cells differ between the fovea and midperiphery. Thirdly, although the approximate injection volumes were determined using the area of retinal detachment, the injected volumes were not necessarily the same at each injection site. In future studies, it will be necessary to adjust the injection volumes of all retinal detachments, which could be made possible by monitoring the injection volume with intraoperative OCT. Fourthly, the time point to evaluate photoreceptor cell death by TUNEL was limited to 1 week after injections due to the number of available monkeys. Considering the peak of TUNEL staining in previous reports on retinal detachment in animal models [35,36], evaluation at an earlier time point, such as 1–3 days after injection, will be necessary in future studies. Finally, the present study examined the influence of injection pressure on retina histology, but the influence of injection pressure on retinal function remains unknown. Future studies are needed to investigate functional changes in the retina after damage to the retinal outer layer and RPE as well as after their recovery.

In summary, our results show that the photoreceptor layer and RPE can be damaged by subretinal injection and that the degree of damage depends on the injection pressure. To perform subretinal injection safely, clinicians must inject at the lowest possible pressure.

## Supporting information

S1 Fig The sites of subretinal injections and the areas of retinal detachment in the monkey eye-1. A and B: The same fundus picture taken 3 weeks after subretinal injection. In B, the area of internal limiting membrane removal (a) and areas of retinal detachment due to subretinal injection (b–e) are illustrated as circles. Cross marks indicate the sites of subretinal injection at b-e. C–E: B-scan optical coherence tomography (OCT) images captured at “a”. F–H: B-scan OCT images captured at “b”. I–K: B-scan OCT images captured at “c”. L–N: B-scan OCT images captured at “d”. O–Q: B-scan OCT images captured at “e”. OCT images of both the control (removal of the ILM without subretinal injection of balanced salt solution; BSS) and minimum-pressure groups (BSS injection at 6 psi) show a well-preserved retinal structure, including continuity of the ellipsoid zone (EZ) throughout the experimental period (C–N). OCT images of the high-pressure group (BSS injection at 20 psi) show EZ disruption at 1 week after injection (asterisk in O). At 3 weeks after injection, OCT images show partial recovery of the EZ (asterisk in P). The EZ finally became continuous at 5 weeks after injection (asterisk in Q). The eye was enucleated 6 weeks after subretinal injections and used for light and transmission electron microscopy. Scale bars = 200 μm.
(TIF)

S2 Fig The sites of subretinal injections and the areas of retinal detachment in the monkey eye-2. A and B: The same fundus picture taken 4 weeks after subretinal injection. In B, the areas of internal limiting membrane removal (a and b) and the areas of retinal detachment due to subretinal injection (c–f) are illustrated as circles. Cross marks indicate the sites of subretinal injection at c–f. C–E: B-scan optical coherence tomography (OCT) images captured at “a”. F–H: B-scan OCT images captured at “b”. I–K: B-scan OCT images captured at “c”. L–N: B-scan OCT images captured at “d”. O–Q: B-scan OCT images captured at “e”. R–T: B-scan OCT images captured at “f”. OCT images of both control (no injection of balanced salt solution; BSS) and minimum-pressure (BSS injection at 6 psi) groups show a well-preserved retinal structure throughout the experimental period, including continuity of the ellipsoid zone (EZ) (C to Q). OCT images of the high-pressure group (BSS injection at 20 psi) show EZ disruption at 1 week after injection (asterisk in R). The EZ became continuous at 3 and 5 weeks after injection (asterisks in S and T). The eye was enucleated 6 weeks after subretinal injections and used for TdT-dUTP terminal nick-end labeling. Scale bars = 200 μm.
(TIF)

S3 Fig The sites of subretinal injections and the areas of retinal detachment in the monkey eye-3. A and B: The same fundus picture taken 1 week after subretinal injection. In B, the areas of retinal detachment due to subretinal injection (a–e) are illustrated as circles. Cross marks indicate the sites of subretinal injection at a–e. C: B-scan optical coherence tomography (OCT) images captured at “a”. D: B-scan OCT images captured at “b”. E: B-scan OCT images captured at “c”. F: B-scan OCT images captured at “d”. G: B-scan OCT images captured at “e”. OCT images of minimum-pressure (BSS injection at 6 psi) group show a well-preserved retinal structure at 1 week after injection, including continuity of the ellipsoid zone (EZ) (C–E). OCT images of the high-pressure group (BSS injection at 20 psi) show EZ disruption at 1 week after injection (asterisks in F and G). The eye was enucleated 1 week after subretinal injections and used for light and transmission electron microscopy. Scale bars = 200 μm.
(TIF)

S4 Fig The sites of subretinal injections and the areas of retinal detachment in the monkey eye-4. A and B: The same fundus picture taken 1 week after subretinal injection. In B, the areas of retinal detachment due to subretinal injections (a–f) are illustrated as circles. Cross marks indicate the sites of subretinal injection at a–f. C: B-scan optical coherence tomography (OCT) images captured at “a”. D: B-scan OCT images captured at “b”. E: B-scan OCT images captured at “c”. F: B-scan OCT images captured at “d”. G: B-scan OCT images captured at “e”. H: B-scan OCT images captured at “f”. OCT images of minimum-pressure (BSS injection at 6 psi) group show a well-preserved retinal structure at 1 week after injection, including continuity of the ellipsoid zone (EZ) (C to F). OCT images of the high-pressure group (BSS injection at 20 psi) show EZ disruption at 1 week after injection (asterisk in G and H). The eye was enucleated 1 week after subretinal injections and used for TdT-dUTP terminal nick-end labeling. Scale bars = 200 μm.
(TIF)

S5 Fig Positive control of TdT-dUTP terminal nick-end labeling. Lymph nodes taken simultaneously at the time of enucleation of monkey eyes were used as the positive control for TdT-dUTP terminal nick-end labeling (TUNEL). Arrows show TUNEL-positive cells. Scale bar = 20 μm.
(TIF)
