# Supplementary figures and images for: The influence of subretinal injection pressure on the microstructure of the monkey retina
Source: PLoS One. 2018 Dec 31;13(12):e0209996. doi: 10.1371/journal.pone.0209996 (PMC6312337; doi:10.1371/journal.pone.0209996)

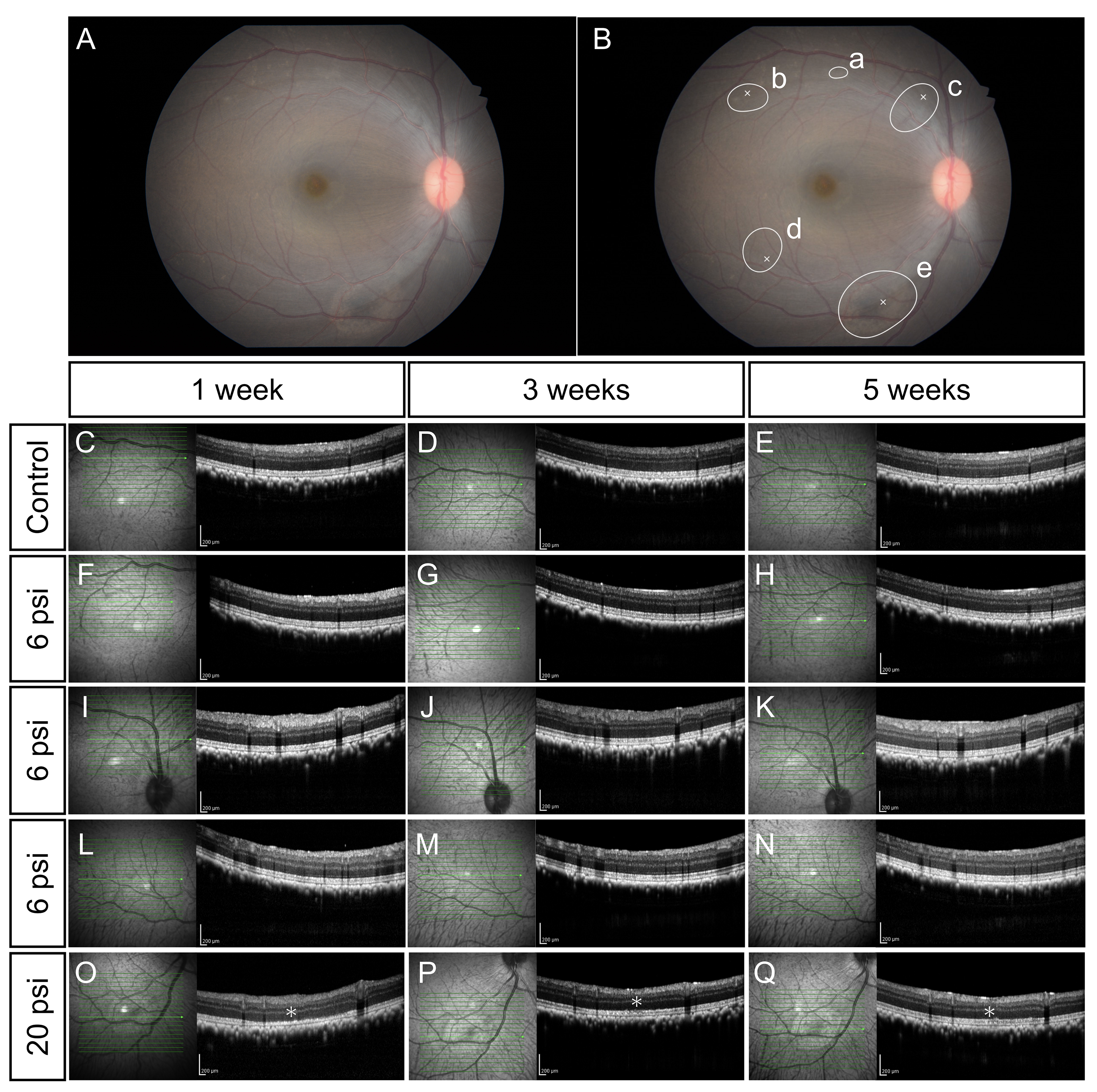

Supplement: S1 Fig — A and B: The same fundus picture taken 3 weeks after subretinal injection. In B, the area of internal limiting membrane removal (a) and areas of retinal detachment due to subretinal injection (b–e) are illustrated as circles. Cross marks indicate the sites of subretinal injection at b-e. C–E: B-scan optical coherence tomography (OCT) images captured at “a”. F–H: B-scan OCT images captured at “b”. I–K: B-scan OCT images captured at “c”. L–N: B-scan OCT images captured at “d”. O–Q: B-scan OCT images captured at “e”. OCT images of both the control (removal of the ILM without subretinal injection of balanced salt solution; BSS) and minimum-pressure groups (BSS injection at 6 psi) show a well-preserved retinal structure, including continuity of the ellipsoid zone (EZ) throughout the experimental period (C–N). OCT images of the high-pressure group (BSS injection at 20 psi) show EZ disruption at 1 week after injection (asterisk in O). At 3 weeks after injection, OCT images show partial recovery of the EZ (asterisk in P). The EZ finally became continuous at 5 weeks after injection (asterisk in Q). The eye was enucleated 6 weeks after subretinal injections and used for light and transmission electron microscopy. Scale bars = 200 μm. (TIF) [file pone.0209996.s001.tif]

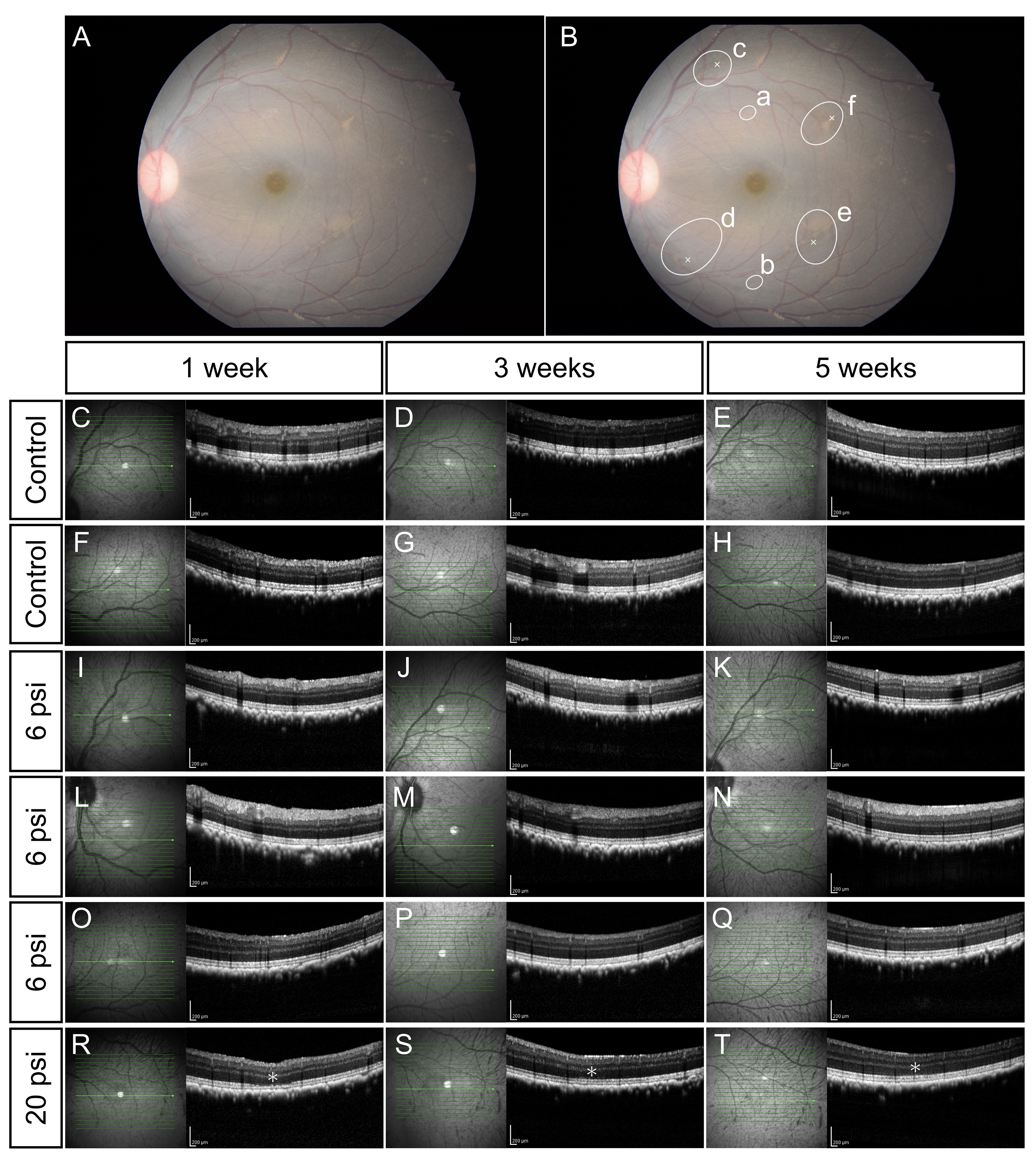

Supplement: S2 Fig — A and B: The same fundus picture taken 4 weeks after subretinal injection. In B, the areas of internal limiting membrane removal (a and b) and the areas of retinal detachment due to subretinal injection (c–f) are illustrated as circles. Cross marks indicate the sites of subretinal injection at c–f. C–E: B-scan optical coherence tomography (OCT) images captured at “a”. F–H: B-scan OCT images captured at “b”. I–K: B-scan OCT images captured at “c”. L–N: B-scan OCT images captured at “d”. O–Q: B-scan OCT images captured at “e”. R–T: B-scan OCT images captured at “f”. OCT images of both control (no injection of balanced salt solution; BSS) and minimum-pressure (BSS injection at 6 psi) groups show a well-preserved retinal structure throughout the experimental period, including continuity of the ellipsoid zone (EZ) (C to Q). OCT images of the high-pressure group (BSS injection at 20 psi) show EZ disruption at 1 week after injection (asterisk in R). The EZ became continuous at 3 and 5 weeks after injection (asterisks in S and T). The eye was enucleated 6 weeks after subretinal injections and used for TdT-dUTP terminal nick-end labeling. Scale bars = 200 μm. (TIF) [file pone.0209996.s002.tif]

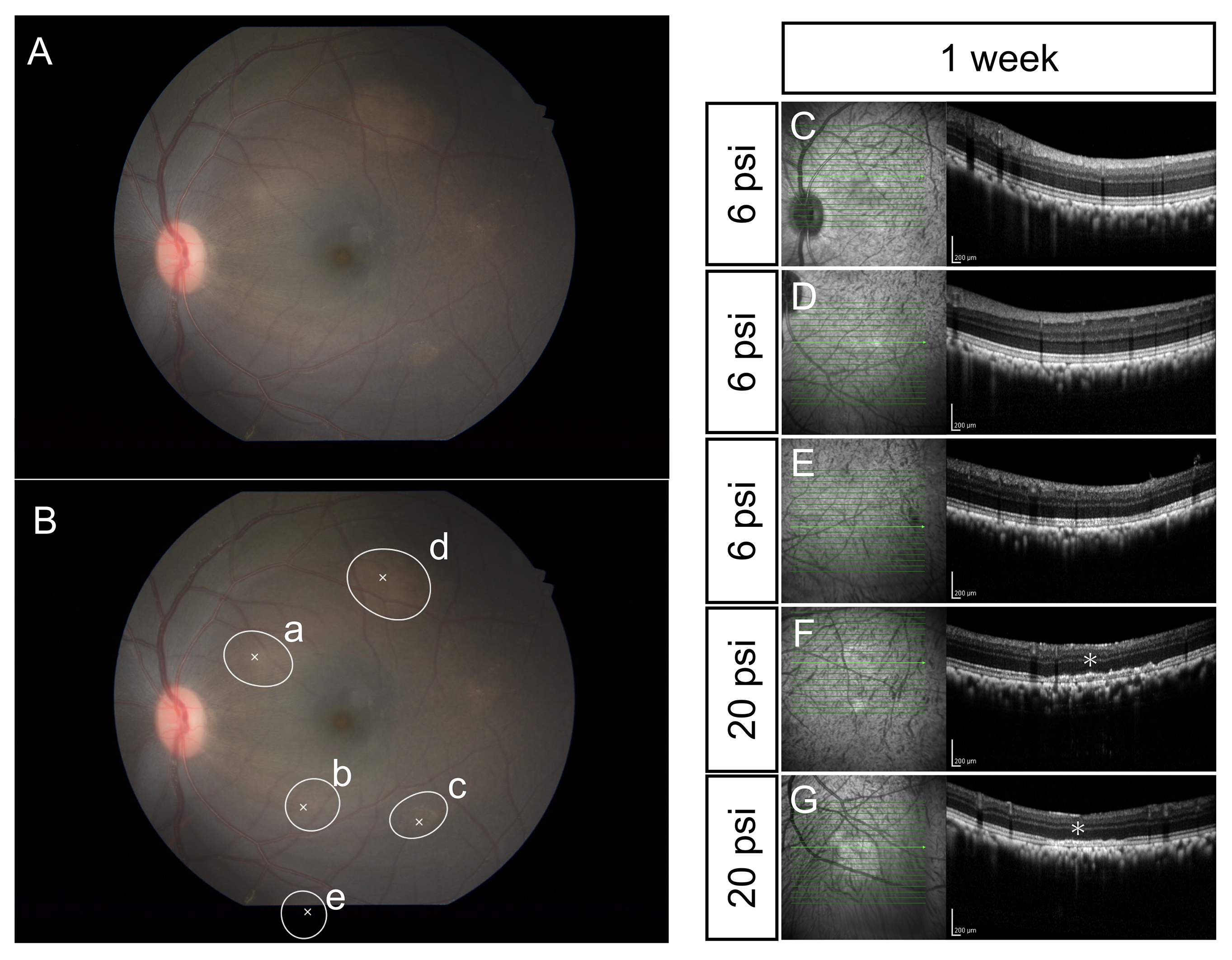

Supplement: S3 Fig — A and B: The same fundus picture taken 1 week after subretinal injection. In B, the areas of retinal detachment due to subretinal injection (a–e) are illustrated as circles. Cross marks indicate the sites of subretinal injection at a–e. C: B-scan optical coherence tomography (OCT) images captured at “a”. D: B-scan OCT images captured at “b”. E: B-scan OCT images captured at “c”. F: B-scan OCT images captured at “d”. G: B-scan OCT images captured at “e”. OCT images of minimum-pressure (BSS injection at 6 psi) group show a well-preserved retinal structure at 1 week after injection, including continuity of the ellipsoid zone (EZ) (C–E). OCT images of the high-pressure group (BSS injection at 20 psi) show EZ disruption at 1 week after injection (asterisks in F and G). The eye was enucleated 1 week after subretinal injections and used for light and transmission electron microscopy. Scale bars = 200 μm. (TIF) [file pone.0209996.s003.tif]

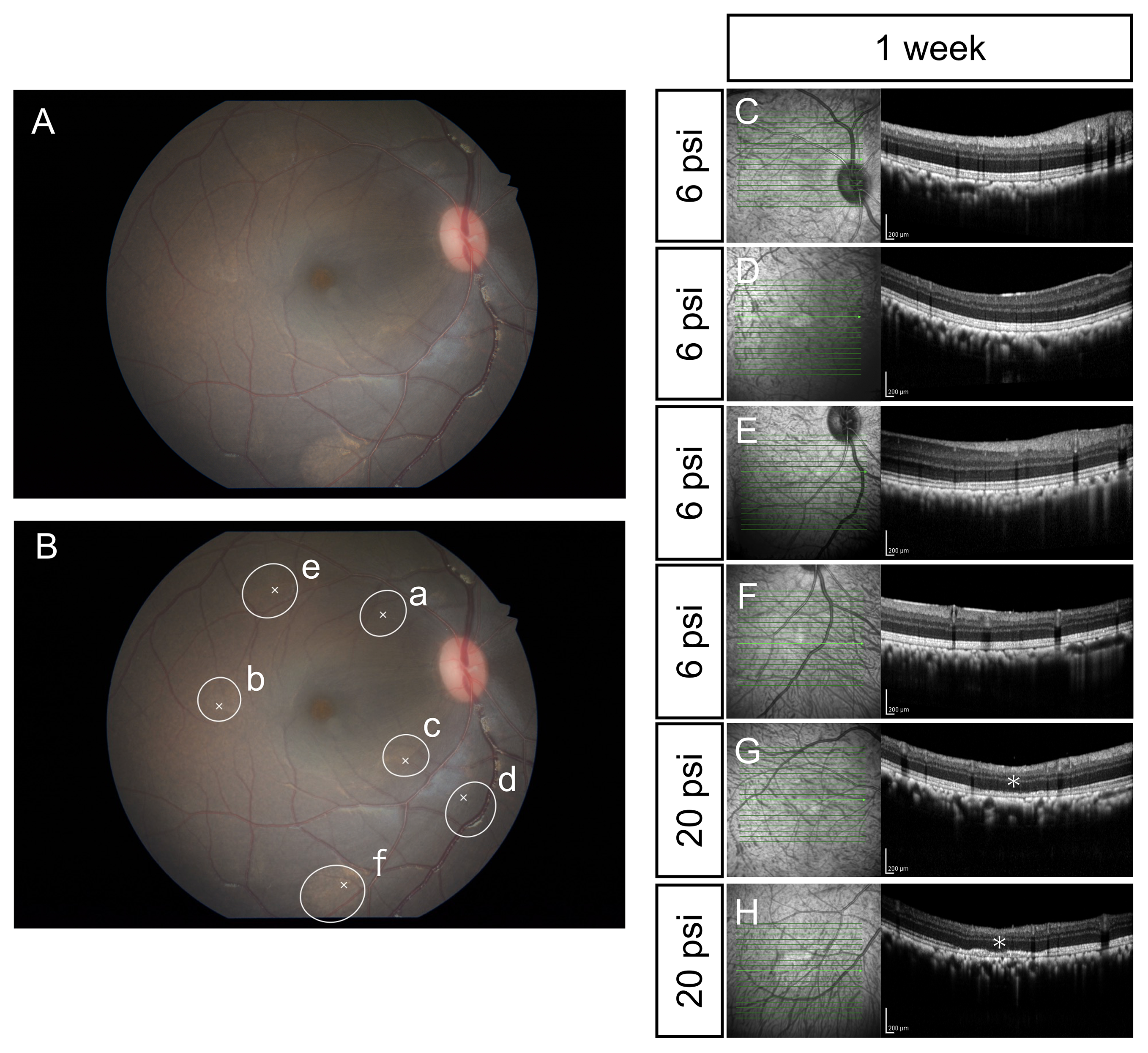

Supplement: S4 Fig — A and B: The same fundus picture taken 1 week after subretinal injection. In B, the areas of retinal detachment due to subretinal injections (a–f) are illustrated as circles. Cross marks indicate the sites of subretinal injection at a–f. C: B-scan optical coherence tomography (OCT) images captured at “a”. D: B-scan OCT images captured at “b”. E: B-scan OCT images captured at “c”. F: B-scan OCT images captured at “d”. G: B-scan OCT images captured at “e”. H: B-scan OCT images captured at “f”. OCT images of minimum-pressure (BSS injection at 6 psi) group show a well-preserved retinal structure at 1 week after injection, including continuity of the ellipsoid zone (EZ) (C to F). OCT images of the high-pressure group (BSS injection at 20 psi) show EZ disruption at 1 week after injection (asterisk in G and H). The eye was enucleated 1 week after subretinal injections and used for TdT-dUTP terminal nick-end labeling. Scale bars = 200 μm. (TIF) [file pone.0209996.s004.tif]

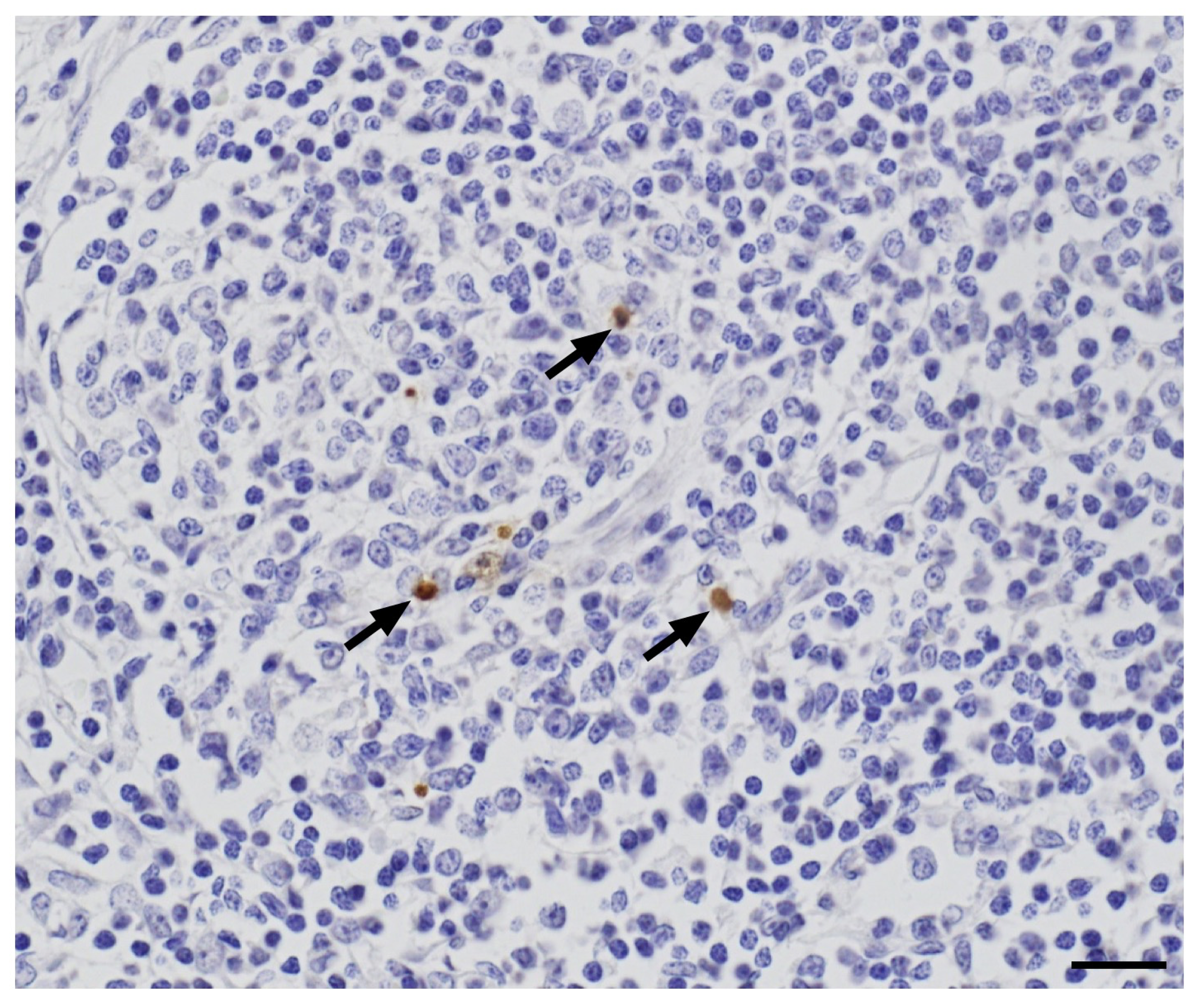

Supplement: S5 Fig — Lymph nodes taken simultaneously at the time of enucleation of monkey eyes were used as the positive control for TdT-dUTP terminal nick-end labeling (TUNEL). Arrows show TUNEL-positive cells. Scale bar = 20 μm. (TIF) [file pone.0209996.s005.tif]
